# Association of the ANCA Kidney Risk Score With Kidney Failure in Anti-Glomerular Basement Membrane Disease

**DOI:** 10.1016/j.ekir.2026.107130

**Published:** 2026-10-06

**Authors:** Pauline Caillard, Cécile Vigneau, Jean-Michel Halimi, Marc Hazzan, Eric Thervet, Morgane Heitz, Laurent Juillard, Vincent Audard, Marion Rabant, Alexandre Hertig, Benoit Brilland, Vincent Vuiblet, Dominique Guerrot, Mathilde Tamain, Marie Essig, Thierry Lobbedez, Thomas Quemeneur, Mathieu Legendre, Alexandre Ganea, Marie-Noëlle Peraldi, François Vrtovsnik, Maïté Daroux, Gabriel Choukroun, Dimitri Titeca-Beauport

**Affiliations:** 1 Department of Nephrology, Dialysis and Transplantation, Limoges Medical Center, Limoges, France; 2 CRIBL Laboratory, UMR CNRS 7276 INSERM 1262, University of Limoges, Limoges, France; 3 Division of Nephrology, CHU Rennes, Inserm, EHESP, Irset (Institut de Recherche en Santé, Environnement et Travail), UMR_S, 1085, Rennes University Hospital, University of Rennes, Rennes, France; 4 Department of Nephrology, Tours University Hospital and EA4245, University of Tours, Tours, France; 5 Nephrology Department, Lille University Hospital, University of Lille, UMR 995, Lille, France; 6 Department of Nephrology, Georges Pompidou European Hospital, Assistance Publique-Hôpitaux de Paris (APHP), INSERM UMRS970, Boulogne-Billancourt, France; 7 Department of Nephrology and Dialysis, Annecy Genevois Hospital, Pringy, France; 8 Department of Nephrology, Hospital Croix-Rousse, Hospices Civils de Lyon, Carmen INSERM 1060, University Lyon 1, Lyon, France; 9 Department of Nephrology and Renal Transplantation, Reference Center-Idiopathic Nephrotic Syndrome, Hôpitaux Universitaires Henri Mondor, APHP, INSERMU955, Paris Est Créteil University, Créteil, France; 10 Pathology Department, Necker University Hospital, AP-HP, Centre-Université de Paris, Paris, France; 11 Department of Nephrology, Dialysis and Transplantation, Foch Hospital, Versailles Saint-Quentin University, Suresnes, France; 12 Department of Nephrology, Dialysis and Transplantation, University Hospital, Angers and CRCINA, INSERM, Nantes University, Angers University, Angers, France; 13 Department of Nephrology and Renal Transplantation, Reims University Hospital, Reims, France; 14 Department of Nephrology, Rouen University Hospital, Rouen and INSERM, Rouen, France; 15 Department of Nephrology and Dialysis, Vichy Hospital, Vichy, France; 16 Department of Nephrology, Dialysis, and Renal Transplantation, Ambroise-Paré Hospital, AP-HP, Paris-Saclay University, Boulogne-Billancourt, France; 17 Department of Nephrology, Caen University Hospital, Caen, France and the French Registry of Peritoneal Dialysis, Langue Française, Pontoise, France; 18 Department of Nephrology and Internal Medicine, Valenciennes General Hospital, Valenciennes, France; 19 Department of Nephrology, Dialysis and Renal Transplantation, University Hospital, Dijon, France; 20 Department of Nephrology, Orleans Hospital, Orleans, France; 21 Department of Nephrology, Dialysis and Renal Transplantation, Centre-Université de Paris, Necker University Hospital, AP-HP, Paris, France; 22 Nephrology Department, Faculty of Medicine, Bichat-Claude Bernard Hospital, APHP, Paris Diderot University, Sorbonne Paris Cité, Paris, France; 23 Department of Nephrology, Duchenne Hospital, Boulogne-Sur-Mer, France; 24 Department of Nephrology, Dialysis, and Transplantation, University of Picardie Jules Verne, Amiens University Hospital, Amiens, France; 25 Mécanismes Physiopathologiques et Conséquences des Calcifications Cardiovasculaires (MP3CV) Laboratory, Centre de Recherche en Santé (CURS), Amiens, France

**Keywords:** anti-GBM disease, ANCA-associated vasculitis, AKRiS, double-positive patients, kidney survival

## Abstract

**Introduction:**

Anti-glomerular basement membrane (anti-GBM) disease is a rare vasculitis that is characterized by a poor renal prognosis. Despite the high predictive accuracy of the improved antineutrophil cytoplasmic autoantibody (ANCA) Kidney Risk Score (AKRiS) in ANCA-associated vasculitis, its prognostic value in anti-GBM disease remains to be established.

**Methods:**

In this multicenter retrospective study, 163 biopsy-proven patients with anti-GBM were included. AKRiS was calculated at diagnosis, stratifying patients into low-, moderate-, high-, and very high-risk groups. The Fine-Gray model was used for the analysis of 36-month kidney failure, with the predictive performance assessed via Harrell C-index.

**Results:**

The median AKRiS was 18 [11–21], with 69% of patients classified as high or very high-risk. The 36-month cumulative incidence of kidney failure was significantly higher in the high-risk (92% [95% confidence interval (CI), 82–97]) and very high-risk (98% [95% CI, 86–100]) groups, when compared with the low-risk (40% [95% CI, 19–61]; *P* < 0.0001). In the multivariable analysis, AKRiS was independently associated with a poor renal prognosis (subhazard ratio [sHR] 2.51 and 2.82 for the high- and very high-risk groups vs. low-risk, respectively). The model combining oligoanuria and AKRiS improved the predictive performance of AKRiS alone (C-index 0.90 [95% CI, 0.85–0.94] vs. 0.85 [95% CI, 0.80–0.91]; *P* = 0.02). Despite higher AKRiS scores, double-positive patients had similar 36-month kidney failure compared with single-positive patients, reflecting their divergent pathophysiological and therapeutic responsiveness.

**Conclusion:**

AKRiS is associated with renal risk in anti-GBM disease, irrespective of ANCA status. Its predictive performance further improves when combined with oligoanuria, supporting the development of pragmatic prognostic models for anti-GBM disease.

## Graphical abstract

**Figure:**
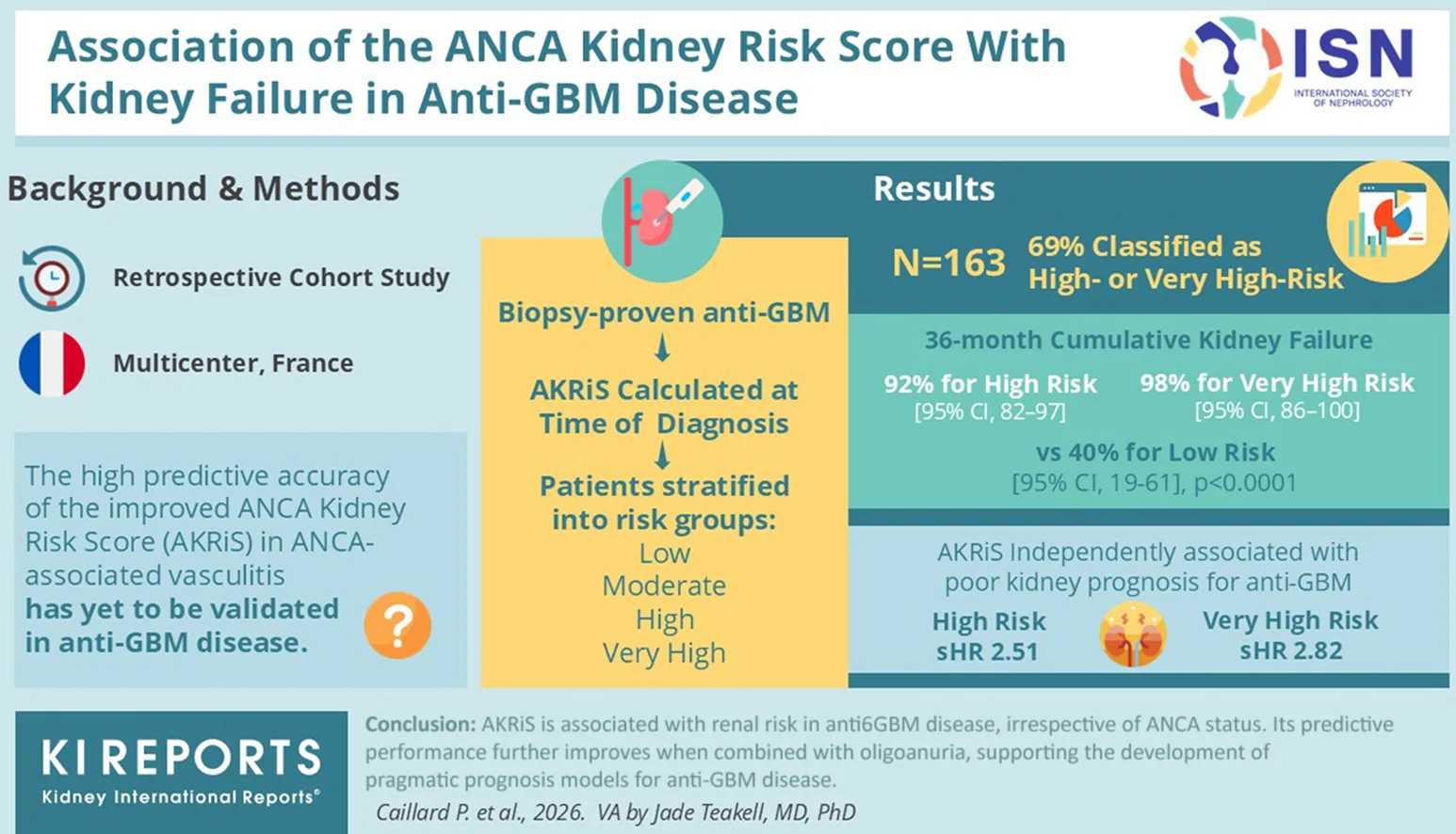

Anti-GBM disease is a rare but highly aggressive form of small-vessel vasculitis.^1^, ^2^ It is usually characterized by circulating anti-GBM antibodies resulting in rapidly progressive glomerulonephritis and/or diffuse alveolar hemorrhage.^3^, ^4^ Between 20% and 40% of patients with anti-GBM disease are also positive for ANCA, most often with a myeloperoxidase specificity.^5^ These patients present clinical and biological features that overlap with both ANCA-associated vasculitis (AAV) and classic anti-GBM disease, with a kidney survival close to anti-GBM but lower than in patients with AAV.^6^, ^7^, ^8^, ^9^, ^10^, ^11^ In both single- (SPPs) and double-positive (DPPs) patients with anti-GBM, several baseline factors are strongly associated with poor kidney prognosis, including elevated serum creatinine (SCr ≥ 500 μmol/l), oligoanuria, dialysis requirement at diagnosis, and a high percentage of crescents on kidney biopsy (≥ 85%).^3^, ^11^, ^12^, ^13^ As a result, several studies have used kidney histologic parameters, including glomerular lesions as categorized by the Berden classification, and markers of tubulo-interstitial injury (such as interstitial inflammation, fibrosis, and tubular atrophy), to predict kidney outcomes in anti-GBM disease.^14^, ^15^, ^16^, ^17^ In the cohort reported by Van Daalen *et al.*,^17^ the percentage of normal glomeruli and the degree of interstitial infiltrate, combined with dialysis dependency at diagnosis, were better predictors of kidney failure than the Berden classification in patients with anti-GBM. These findings highlighted the need for histology-based prognostic tools specifically applicable to anti-GBM disease. Over recent years, Brix *et al.*^18^ developed a new histopathologic classification for AAV to better predict kidney outcomes, known as the ANCA Renal Risk Score (ARRS). This score integrated 3 of the following variables: baseline estimated glomerular filtration rate (eGFR), the percentage of normal glomeruli, and the extent of interstitial fibrosis and/or tubular atrophy (IFTA). Although initially designed for AAV, Floyd *et al.*^7^ applied this classification in a retrospective study of 174 patients with anti-GBM, and demonstrated its applicability in this population and ability to predict kidney survival. The percentage of normal glomeruli and the need for dialysis at diagnosis were the best predictors of kidney survival.^7^, ^19^ In 2024, the ARRS was revised to increase its prognostic accuracy and applicability, leading to the development of the improved AKRiS.^20^ This updated version standardized the scoring criteria by replacing the eGFR with SCr as the renal parameter, and by refining the evaluation of histologic parameters, such as the IFTA score and the percentage of normal glomeruli. Although the use of SCr may introduce age- and sex-related bias compared with eGFR, particularly in women, these modifications enhanced the reliability of disease activity assessment, improved prognostic discrimination of kidney outcomes in AAV, and facilitated routine clinical use.

This study aimed to assess the performance of the AKRiS at the time of diagnosis to predict kidney outcomes in SPPs and DPPs anti-GBM disease.

## Methods

### The Study Cohort

We conducted a retrospective multicenter study. The study protocol was approved by the local independent institutional review board (CPP Nord Ouest II, Amiens, France; reference: TB/LR/2016-91).

Data from 208 patients diagnosed with anti-GBM disease were retrospectively extracted from the medical records of 22 French Nephrology Departments (19 university and 3 tertiary hospitals) from January 1997 to December 2017. Anti-GBM disease was defined as glomerulonephritis with crescent formation and linear glomerular IgG deposits on renal biopsy, regardless of serum anti-GBM antibody positivity. Seronegative anti-GBM disease was defined as patients with biopsy-proven anti-GBM disease but no detectable circulating antibodies. Patients with anti-GBM disease and concurrent positive ANCA assay results (indirect immunofluorescence and/or antigen-specific immunoassay specific for myeloperoxidase or proteinase 3) were also identified as a DPP subgroup. The time of diagnosis was defined as the date on which anti-GBM antibodies were first detected in serum and/or on kidney biopsy.

### Clinical, Biological, and Histologic Data

Clinical and laboratory data at diagnosis and during follow-up were retrieved from each center’s medical records. Light microscopy (percentage of crescents and presence of tubular and interstitial damage) and immunofluorescence (type and location of Ig and/or complement depositions) findings on kidney biopsies were recorded. All biopsies were rated according to the ARRS and revised AKRiS.^18^, ^20^ Different cutoff values were used for the ARRS parameters, as follows: eGFR at diagnosis (G0 > 15 ml/min per 1.73 m^2^, G1 ≤ 15 ml/min per 1.73 m^2^), the percentage of normal glomeruli (N0 > 25%, N1 10 to 25%, N2 < 10%), the percentage of IFTA (T0 ≤ 25%, T1 > 25%), and defined 3 risk groups: low (0 point (pt)), medium (2–7 pts), and high (8–11 pts). The AKRiS was also based on the following 3 parameters with specific scoring: SCr (< 250 μmol/l = 0 pt, 250–450 μmol/l = 4 pts, > 450 μmol/l = 11 pts), the percentage of normal glomeruli (> 25% = 0 pt, 10%–25% = 4 pts, < 10% = 7 pts), and IFTA (none/mild or < 25% = 0 pt, mild-moderate or ≥ 25% = 3 pts). Based on this score, 4 risk groups were established: low (0–4 pts), moderate (5–11 pts), high (12–18 pts), and very high (21 pts). SCr corresponded to the highest stable value measured at diagnosis, before initiation of immunosuppressive therapy and, when applicable, before dialysis initiation. Normal glomeruli were defined as glomeruli without crescents, necrosis, or global sclerosis, in accordance with previously published classifications.^20^ AKRiS was intended to be applied at the time of diagnosis, using variables reflecting baseline renal function and histologic damage, such as in AAV.^20^ Oligoanuria was defined at time of diagnosis by a urine output that is less than 500 ml per day.

Treatment regimens were categorized as intensive treatment, defined as the combination of corticosteroids, plasma exchanges (PLEX), and immunosuppressive agents (cyclophosphamide and/or rituximab), or alternative treatment, comprising any 2 of corticosteroids, immunosuppressive agents, or PLEX; or corticosteroids alone; or supportive care. The need for dialysis during initial presentation referred to the requirement for dialysis during the initial hospital stay. Alive and kidney failure-free patients were defined as patients who remained alive without kidney transplantation or dialysis-dependence at 12 and 36 months of follow-up.

Kidney failure was defined as the need for dialysis for at least 12 weeks after initiation. The date of kidney failure was defined as the first day of dialysis initiation. Patients who died before completing 12 weeks of dialysis were classified as having reached kidney failure if they remained dialysis-dependent until death, in order to avoid immortal time bias. Patient survival was defined as the time interval between diagnosis and death. The date of the last follow-up corresponded to the patient’s death or the last visit before the end of the study (December 31, 2018). The primary end point was the time to kidney failure over a 36-month period, with death treated as competing event.

### Statistical Analysis

Patient characteristics were summarized as frequencies (%) for categorical variables and as medians (interquartile range [IQR]) for continuous variables, as most of them were not normally distributed (Shapiro-Wilk test). Categorical variables were compared using a chi-squared test or Fisher exact test, as appropriate. The Kruskal-Wallis test was applied to compare quantitative variables across the 4 risk groups, and the nonparametric Mann-Whitney U test was used for the comparison of SPPs and DPPs. The cumulative incidence of kidney failure was estimated in a competing risks framework, treating death before kidney failure as a competing event. Overall differences between cumulative incidence curves across groups were assessed using Gray test. Additionally, differences in cumulative incidence estimates at specific time points (e.g., 36 months) were evaluated using a Wald-type Z-test based on the estimates and their standard errors. Risk factors for kidney failure were then evaluated using univariable and multivariable Fine-Gray competing-risks regression models. The results were expressed as the sHR (95% CI). The Harrell concordance index (C-index; with 95%CI) evaluated the ability of each model to predict kidney failure.^21^ The threshold for statistical significance was set to *P* < 0.05. All statistical analyses were performed using R software (version 4.5.3, R Foundation for Statistical Computing, Vienna, Austria) in RStudio (version 2025.09.2 + 418, Posit Software, PBC, Boston, MA).

## Results

### Baseline Characteristics of the Study Participants

A total of 208 patients were diagnosed with anti-GBM disease, of whom 163 had an available kidney biopsy, were actively treated and included in the study (Figure 1). Patient clinical and biological characteristics at diagnosis are presented in Supplementary Table S1. The cohort comprised 65 (40%) women and the median (IQR) age at diagnosis was 55 (30–69) years. Chronic arterial hypertension (36%) and tobacco use (40%) were the most common comorbidities. At diagnosis, patients exhibited severe acute kidney failure, with a median (IQR) SCr level of 670 (388–1050) μmol/l, oligoanuria in 104 (67%) patients, and a need for dialysis in 120 (74%) patients during initial presentation. ANCA positivity was found in 47 (29%) patients, defining the DPPs, and 116 (71%) patients had single anti-GBM positivity.

**Figure 1:**
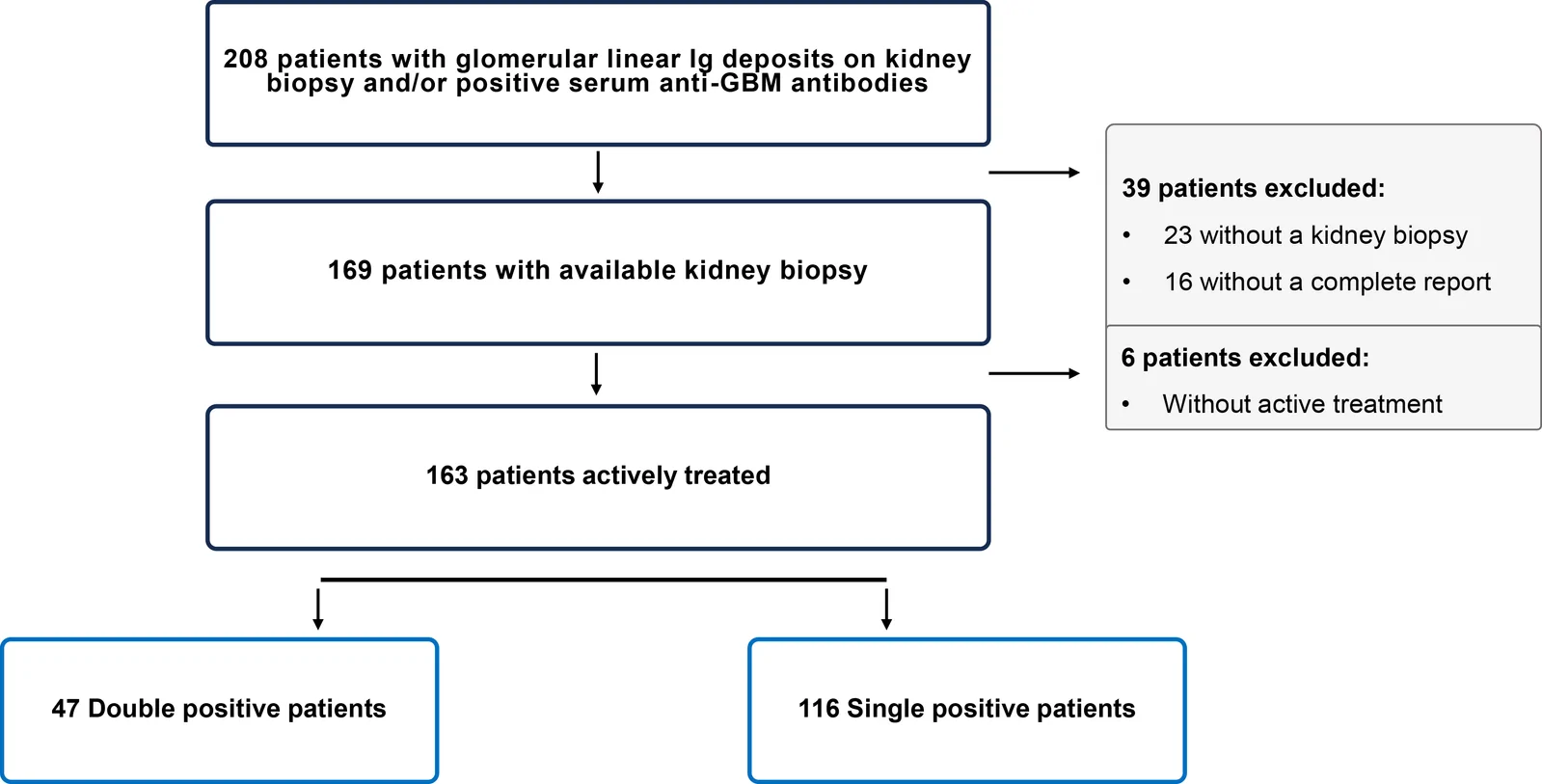
Study flow chart.

Among this cohort, 114 (70%) underwent intensive therapy (Supplementary Table S2). Almost all patients received methylprednisolone pulses, with a median of 3 (3–3) pulses, and 64% were still under corticosteroids at 6 months. PLEX were carried out in 127 (78%) patients, and the most commonly used immunosuppressant was cyclophosphamide (82%).

### Clinical, Biological, and Histologic Characteristics Based on the AKRiS

The median (IQR) AKRiS was 18 (11–21), with high and very high-risk groups accounting for 39% and 30% of the cases, respectively (Table 1). Patients in the high- and very high-risk groups were older (57 [33–72] and 65 [38–76] vs. 38 [23–54] years in the low-risk group; *P* = 0.004; Supplementary Table S1). These patients also exhibited a more severe clinical phenotype, characterized by a greater incidence of oligoanuria (81% and 83% in the high- and very high-risk groups, compared with 5% and 35% in the low- and moderate-risk groups, respectively; *P* < 0.0001) and dialysis requirement at presentation (91% and 98% in the high- and very high-risk groups, respectively, compared with 15% and 39% in the low- and moderate-risk groups, respectively; *P* < 0.0001). None of the patients in the low-risk group tested positive for ANCA, whereas the very high-risk group had the highest positivity rate (46%), compared with 30% and 19% in the high- and moderate-risk groups, respectively (*P* = 0.001). Furthermore, the very high-risk group presented a significantly higher rate of circulating anti-GBM antibody positivity (98%) compared with the low- (75%) and moderate-risk groups (74%; *P* = 0.006). As expected, the severity of the histologic kidney lesions also increased according to the risk group classification (Table 1).

**Table 1:** Kidney biopsy findings at diagnosis

Histological findings	Total (*N* = 163)	Low risk (n = 20)	Moderate risk (n = 31)	High risk (n = 64)	Very high risk (n = 48)	*P*–value
Light microscopy	163 (100)	20 (100)	31 (100)	64 (100)	48 (100)	
Number of normal glomeruli (%)	0 (0–14)	52 (35–75)	0 (0–32)^a^	0 (0–9)^b^^,^^d^	0 (0–0)^c^^,^^e^^,^^f^	< 0.0001
> 25% normal glomeruli	27 (17)	17 (85)	9 (29)^a^	1 (2)^b^^,^^d^	0 (0)^c^^,^^e^	< 0.0001
10–25% normal glomeruli	22 (13)	3 (15)	5 (16)	14 (22)	0 (0)^c^^,^^e^^,^^f^	0.009
˂ 10% normal glomeruli	114 (70)	0 (0)	17 (55)^a^	49 (77)^b^^,^^d^	48 (100)^c^^,^^e^^,^^f^	< 0.0001
Glomerulosclerosis (%)	11 (0–40)	0 (0–0)	11 (0–33)^a^	0 (0–17)^b^	49 (28–75)^c^^,^^e^^,^^f^	< 0.0001
IFTA ≥ 25%	64 (39)	1 (5)	3 (10)	12 (19)^b^	48 (100)^c^^,^^e^^,^^f^	< 0.0001
IFTA (%)^g^	15 (0–40)	0 (0–14)	0 (0–20)	10 [0–20)^b^	50 (40–60)^c^^,^^e^^,^^f^	< 0.0001
AKRiS	18 (11–21)	0 (0–4)	11 (7–11)^a^	18 (18–18)^b^^,^^d^	21 (21–21)^c^^,^^e^^,^^f^	< 0.0001

Values are expressed as the median [interquartile range] or the number (percentage). Anti-GBM, antibodies against glomerular basement membrane; AKRiS, improved ANCA kidney risk score; IFTA, interstitial fibrosis or tubular atrophy.

a *P* < 0.05 low- vs moderate-risk group.

b *P* < 0.05 low- vs high-risk group.

c *P* < 0.05 low- vs very high-risk group.

d *P* < 0.05 moderate- vs high-risk group.

e *P* < 0.05 moderate- vs very high-risk group.

f *P* < 0.05 high- vs very high-risk group.

g n = 158 patients (20 in the low-, 30 in the moderate-, 64 in the high-, and 44 in the very high-risk groups) with the exact percentage of IFTA given on the kidney biopsy reports. The other 5 patients had an IFTA ≥ mild to moderate, considered as ≥ 25%.

### Kidney Failure Cumulative Incidence Based on AKRiS Components

A total of 19 patients (12%) died during follow-up, predominantly in the very high-risk group (Supplementary Table S3). The cumulative incidence of kidney failure was 76.7% (95% CI, 69.4–82.5) at 12 months and 77.9% (95% CI, 70.7–83.6) at 36 months. Only 12 (7%) relapses were observed during follow-up, with a median (IQR) time to onset of 24 (16–67) months.

Figure 2 shows the cumulative incidence of kidney failure at 36 months according to the different AKRiS parameters. Compared with the low-risk group (40.4% [95% CI, 18.8–61.2]), the cumulative incidence of kidney failure at 36 months was significantly higher in the high-risk group (92.2% [95% CI, 81.6–96.8], *P* < 0.001) and the very high-risk group (97.9% [95% CI, 85.8–99.7], *P* < 0.001). No significant difference was observed at this time point between the low-risk and moderate-risk groups (41.9% [95% CI, 24.3–58.6], *P* = 0.91). Regarding the individual parameters of the score, higher baseline SCr levels (K component), the presence of severe tubulointerstitial lesions (T component), and a lower percentage of normal glomeruli (N component) were each associated with a stepwise, statistically significant increase in the 36-month cumulative incidence of kidney failure compared with their respective lowest-risk reference categories (Table 2).

**Figure 2:**
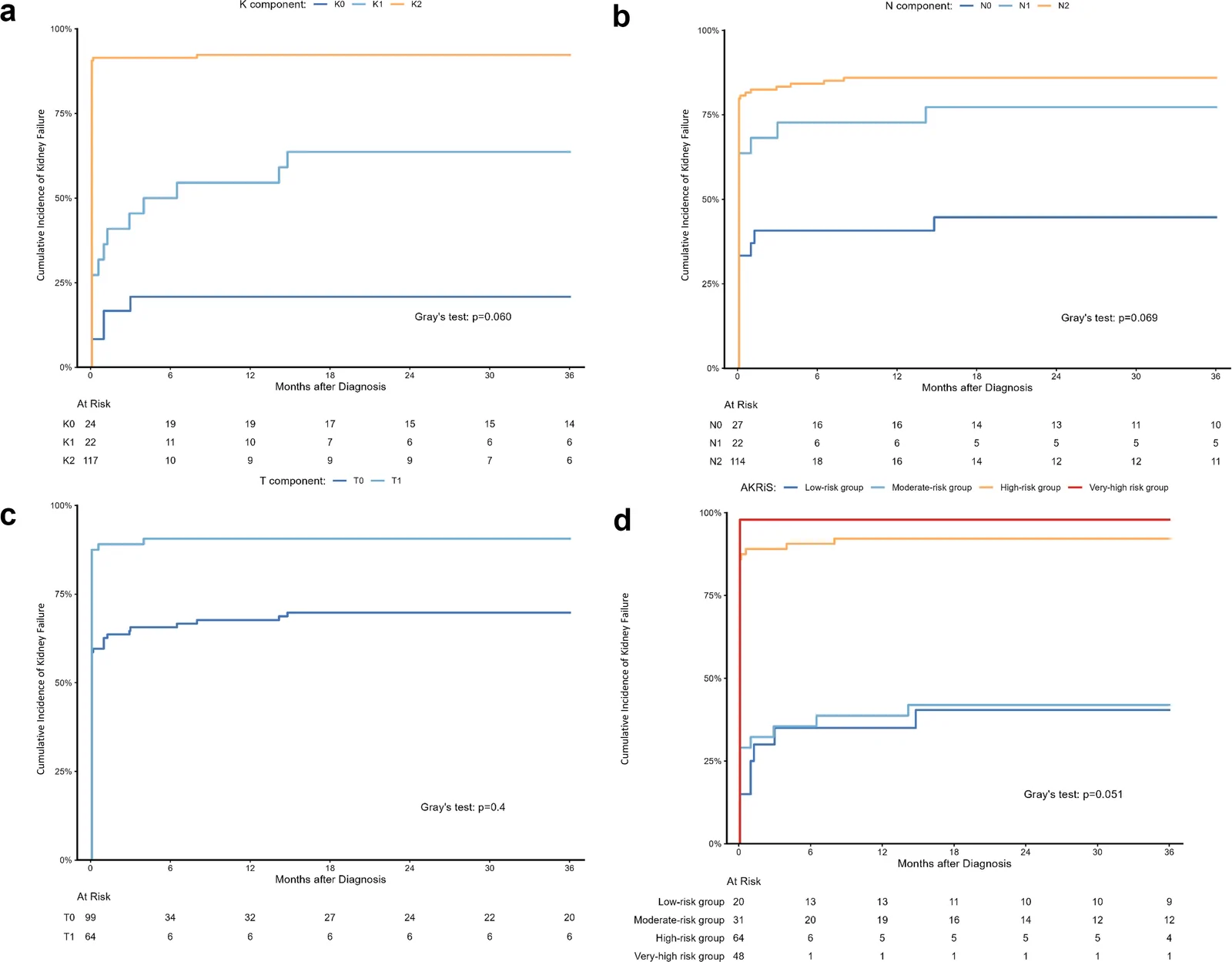
The cumulative incidence curves of 36-month kidney failure stratified by AKRiS in the entire cohort. The curves illustrate the cumulative incidence of kidney failure in the total cohort according to each parameter of the AKRiS: (a) serum creatinine (K component), (b) percentage of normal glomeruli (N component), and (c) interstitial fibrosis and tubular atrophy (T component). Panel (d) shows kidney failure stratified by the four AKRiS risk groups.

**Table 2:** The 36-month cumulative incidence of kidney failure based on AKRiS component

Risk assessment	Category	36-month cumulative incidence (95% CI)	*P*-value (Wald Z-test)
AKRiS global score	Low-risk group	40.4% (18.8%–61.2%)	Reference
	Moderate-risk group	41.9% (24.3%–58.6%)	0.91
	High-risk group	92.2% (81.6%–96.8%)	< 0.001
	Very-high risk group	97.9% (85.8%–99.7%)	< 0.001
K component (creatinine)	K0	20.8% (7.4%–38.9%)	Reference
	K1	63.6% (39.2%–80.4%)	0.002
	K2	92.3% (85.6%–96.0%)	< 0.001
T component (tubulointerstitial)	T0	69.8% (59.6%–77.9%)	Reference
	T1	90.6% (79.9%–95.8%)	< 0.001
N component (normal glomeruli)	N0	44.7% (25.2%–62.4%)	Reference
	N1	77.3% (52.2%–90.3%)	0.017
	N2	86.0% (78.0%–91.2%)	< 0.001

Values are expressed as percentage (%) and 95% confidence interval (95%CI).

### Predictive Performance of the AKRiS for 36-Month Kidney Failure

In the univariable analysis, age at diagnosis, oligoanuria, C-reactive protein, AKRiS, and ARRS were significantly associated with 36-month kidney failure (Table 3). Compared with the ARRS (C-index = 0.78 [95% CI, 0.72–0.85]), the AKRiS demonstrated a significantly better predictive performance, with a C-index of 0.85 (95% CI, 0.80–0.91; *P* = 0.003). When the analysis was limited to the 127 patients who received PLEX, the AKRiS achieved a similar performance with a C-index of 0.84 (95% CI, 0.77–0.90). Furthermore, the predictive performance of the AKRiS did not significantly differ between sexes, yielding a C-index of 0.83 (95% CI, 0.71–0.95) in women and 0.86 (95% CI, 0.80–0.91) in men (*P* = 0.66).

**Table 3:** Factors associated with kidney failure at 36 months in univariable and multivariable Fine-Gray analysis

Variables	Univariable analysis			Multivariable analysis		
	sHR	(95% CI)	*P*-value	sHR	(95% CI)	*P*-value
Age at diagnosis (yr)	1.01	(1.00–1.01)	0.006			
Sex (women)	1.16	(0.95–1.43)	0.2			
Oligoanuria	2.43	(1.75–3.37)	< 0.001	1.57	(1.12–2.19)	0.008
P/C ratio > 3 g/g	1.03	(0.83–1.27)	0.8			
C3 (g/l)	0.80	(0.58–1.10)	0.2			
C4 (g/l)	1.11	(0.31–3.99)	0.9			
CRP (mg/l)	1.00	(1.00–1.00)	< 0.001			
Positive serum ANCA	1.15	(0.92–1.43)	0.2			
Positive serum anti-GBM	1.55	(0.98–2.45)	0.061			
AKRIS			< 0.001			< 0.001
Low-risk group	—	—		—	—	
Moderate-risk group	1.08	(0.50–2.36)		0.96	(0.44–2.11)	
High-risk group	3.39	(1.82–6.30)		2.51	(1.25–5.03)	
Very-high risk group	3.84	(2.08–7.07)		2.82	(1.42–5.63)	
ARRS			< 0.001			
Low-risk group	—	—				
Medium-risk group	1.68	(0.69–4.05)				
High-risk group	3.85	(1.70–8.70)				
Intensive treatment strategy	0.96	(0.76–1.20)	0.7			

ANCA, anti-neutrophil cytoplasmic antigen; Anti-GBM, anti-glomerular basement membrane; AKRiS, improved ANCA kidney risk score; ARRS, ANCA renal risk score; C3 and C4, complement fractions 3 and 4; CRP, c-reactive protein; P/C ratio, urine protein-creatinine ratio.

Values are expressed as subdistribution hazard ratio (sHR) and 95% confidence interval [95%CI].

In the multivariable regression model adjusting for oligoanuria, AKRiS remained an independent predictor of poor kidney outcomes. Compared with the low-risk group, the high- and very high-risk groups exhibited a significantly higher risk of 36-month kidney failure (sHR = 2.51 [95% CI, 1.25–5.03] and sHR = 2.82 [95% CI, 1.42–5.63], respectively). Oligoanuria was also associated with the primary outcome (sHR = 1.57 [95% CI, 1.12–2.19]; *P* = 0.008). Notably, adding this variable significantly improved the predictive performance over AKRiS alone, increasing the C-index from 0.85 (95% CI, 0.80–0.91) to 0.90 (95% CI, 0.85–0.94; *P* = 0.02). Figure 3 illustrates the 36-month cumulative incidence of kidney failure, jointly stratified by AKRiS risk class (moderate, high, very-high) and oligoanuria status, highlighting the additive prognostic impact of oliguria within each risk category (Gray test *P* < 0.001). In the sub-group of patients who received PLEX, this combined model maintained a high predictive accuracy (C-index = 0.87 [95% CI, 0.82–0.93]), although the improvement compared with AKRiS alone was not statistically significant (*P* = 0.06; Supplementary Table S4).

**Figure 3:**
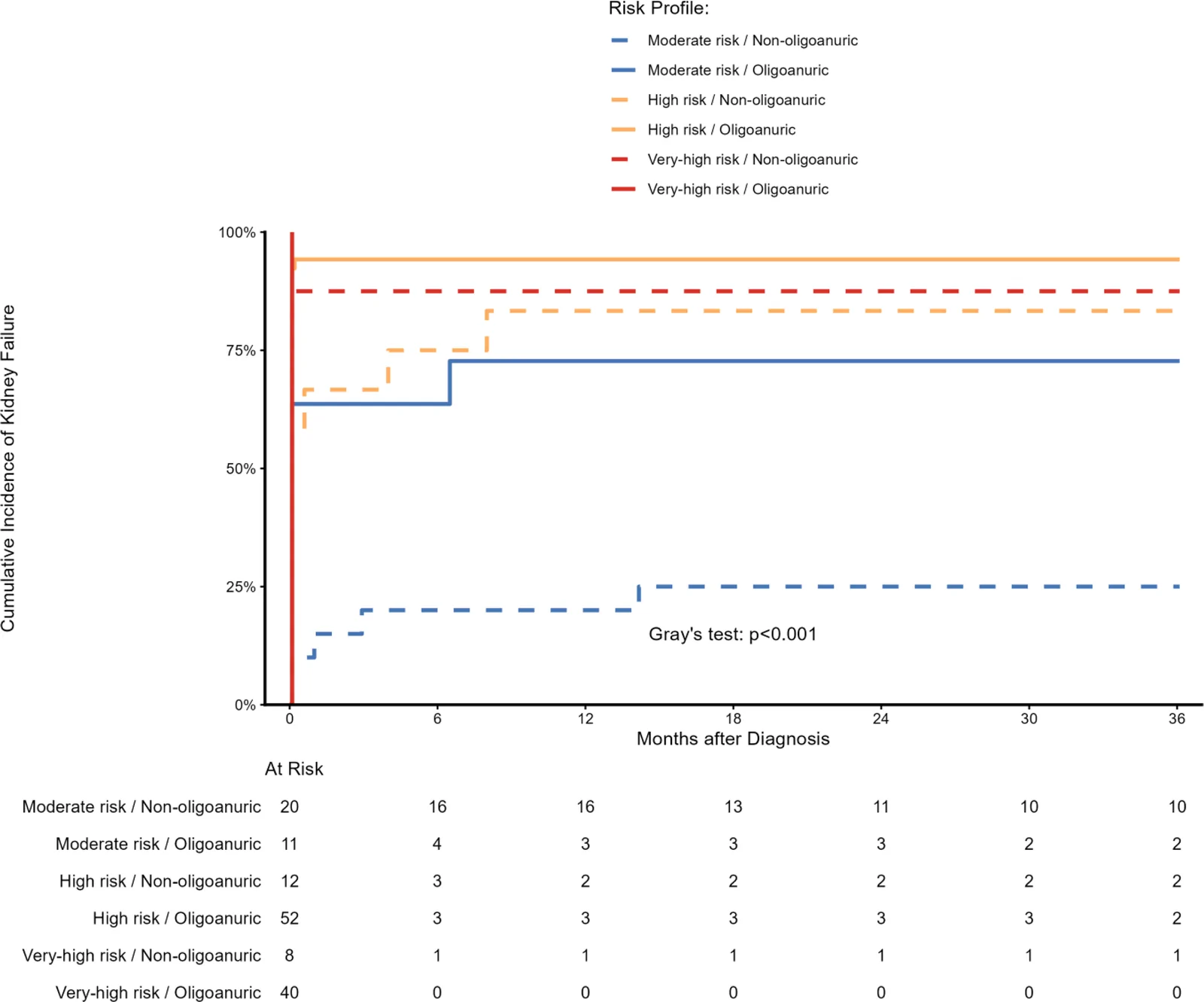
The cumulative incidence curves of 36-month kidney failure stratified by AKRiS and oligoanuria status. The curves illustrate the cumulative incidence of kidney failure in the total cohort according to AKRiS class (moderate, high, and very-high) and oligoanuria status. Patients in the low-risk group were excluded from this analysis because of the presence of only one oligoanuric patient at baseline.

### Comparison of Kidney Failure Between Single- and Double-Positive Patients Based on AKRiS

At presentation, DPPs had more severe kidney involvement, with a higher incidence of oligoanuria (79% vs. 58%; *P* = 0.01), a higher proportion of patients with a SCr > 450 μmol/l (92% vs. 64%; *P* = 0.0004), and a higher need for dialysis (87% vs. 68%; *P* = 0.01) than SPPs (Supplementary Table S5). DPPs also had more chronic histologic kidney lesions affecting both the glomerular (6% of DPPs with more than 25% normal glomeruli vs. 21%, and 33% [0–64] of glomerulosclerosis in DPPs vs. 7% [0–29] respectively; *P* = 0.03 and 0.002), and tubulo-interstitial (62% of DPPs with IFTA ≥ 25% vs. 30%; *P* = 0.0002) compartments compared with the SPPs. Therefore, the AKRiS was higher in DPPs (18 [18–21] vs. 18 [9–18] in SPPs; *P* < 0.0001), with more patients in the very high category (47% vs. 22% of SPPs; *P* = 0.002). There was no difference in therapeutic management between groups.

The cumulative incidence of kidney failure at 36 months was not significantly different between DPPs and SPPs (83% [95% CI, 69–91] vs. 76% [95% CI, 67–83] respectively; *P* = 0.3). Cumulative incidence curves of kidney failure over the first 36 months in SPP and DPP groups are represented in Figure 4. Among the 12 relapses observed, three (6%) occurred in DPPs and were exclusively ANCA-associated relapses (with no anti-GBM recurrence), whereas 9 (8%) occurred in SPPs.

**Figure 4:**
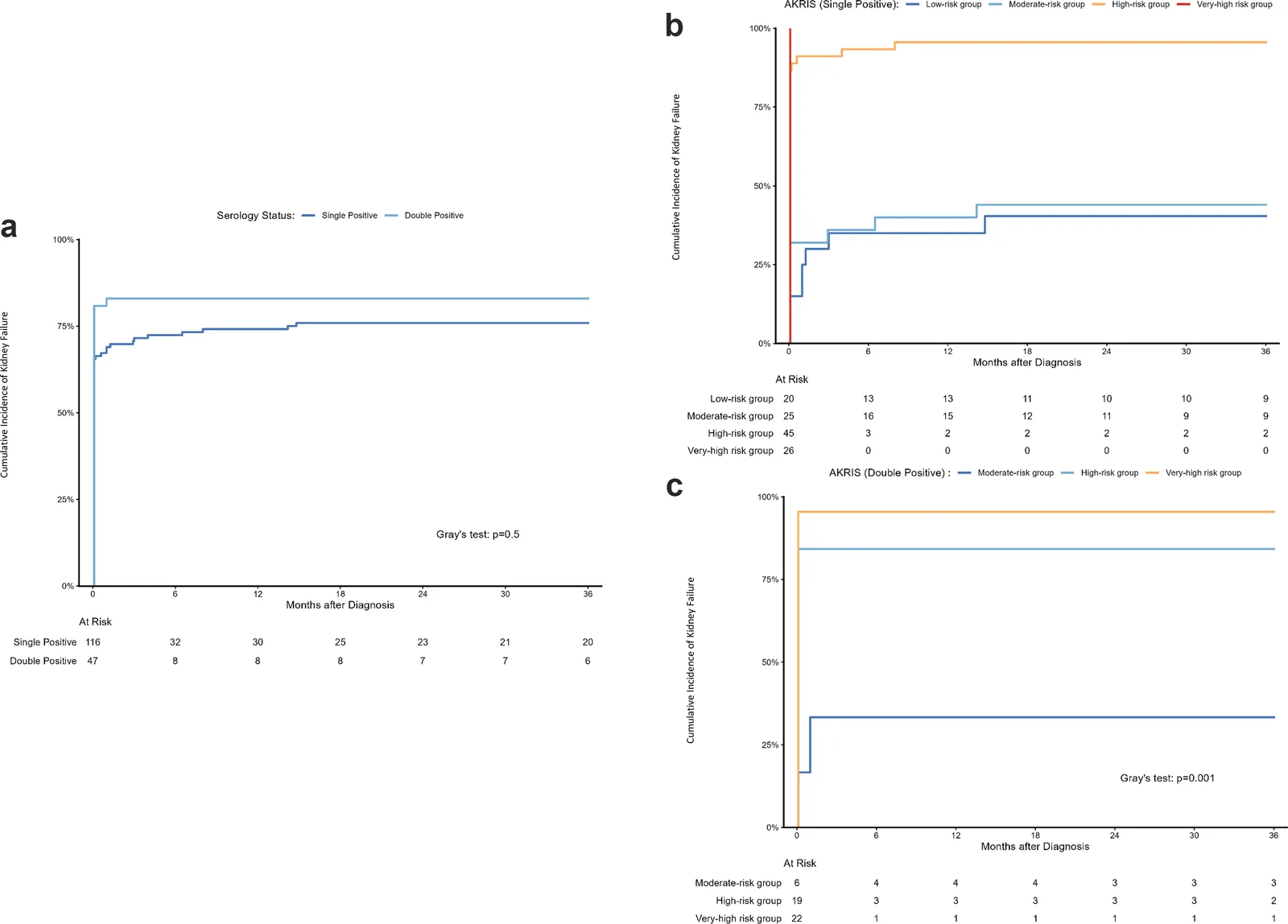
The cumulative incidence curves of 36-month kidney failure stratified by ANCA status. The curves illustrate the cumulative incidence of kidney failure comparing single-positive (SPPs) and double-positive (DPPs) patients (a). Panels (b) and (c) show kidney failure stratified by the four AKRiS risk groups in SPPs and DPPs, respectively. No DPPs were classified in the low-risk group. AKRiS, improved ANCA renal risk score. Gray test was uninformative for the Single Positive subgroup because of early 100% event saturation in the Very-High risk class. Predictive performance is reported via C-index (0.85) and sub-Hazard Ratios (Table 4).

In the univariable analysis, age, oligoanuria, C-reactive protein, AKRiS, and ARRS were associated with 36-month kidney failure in the SPP group, whereas only positive serum anti-GBM and AKRiS were associated with this outcome in DPPs (Table 4). In the DPP group, compared with the moderate-risk group, the high- and very high-risk groups exhibited a higher risk of 36-month kidney failure, although this only reached statistical significance for the very high-risk group (sHR [95% CI], 2.87 [0.94– 8.77] and 3.33 [1.13–9.83], respectively). AKRiS showed a similar predictive performance for 36-month kidney failure in both groups, with a C-index of 0.82 [0.67– 0.97] in DPPs and 0.85 [0.80–0.91] in SPPs (*P* = 0.66). In a multivariable analysis limited to the 116 SPPs, the model combining AKRiS and oligoanuria significantly improved predictive performance over AKRiS alone, increasing the C-index to 0.90 (95% CI, 0.86– 0.95; *P* = 0.015). Supplementary Figure 1 shows the 36-month cumulative incidence of kidney failure stratified by AKRiS risk class and oligoanuria status among SPPs.

**Table 4:** Factors associated with kidney failure at 36 months in double- and single-positive patients

Variables	Single-positive patients (*n* = 116)			Double-positive patients (*n* = 47)		
	sHR	(95% CI)	*P*-value	sHR	(95% CI)	*P*-value
Age at diagnosis (yr)	1.01	(1.00–1.02)	0.003	1.00	(0.99–1.01)	0.7
Sex (women)	1.18	(0.90–1.55)	0.2	1.08	(0.81–1.44)	0.6
Oligoanuria	2.92	(1.94–4.40)	< 0.001	1.58	(0.91–2.74)	0.11
P/C ratio > 3 g/d	1.00	(0.75–1.31)	> 0.9	1.09	(0.82–1.47)	0.5
C3 (g/l)	0.77	(0.52–1.13)	0.2	0.92	(0.57–1.48)	0.7
C4 (g/l)	1.11	(0.24–5.21)	0.9	1.20	(0.14–10.1)	0.9
CRP (mg/l)	1.00	(1.00–1.00)	< 0.001	1.00	(1.00–1.00)	0.3
Positive serum anti-GBM	1.20	(0.74–1.94)	0.5	3.10	(1.04–9.25)	0.042
AKRIS			< 0.001			0.045
Low-risk group	Ref	—		NA	—	
Moderate-risk group	1.15	(0.50–2.65)		Ref	—	
High-risk group	4.00	(2.06–7.76)		2.87	(0.94–8.77)	
Very-high risk group	4.58	(2.40–8.71)		3.33	(1.13–9.83)	
ARRS			< 0.001			0.12
Low-risk group	Ref	—		NA	—	
Medium-risk group	1.71	(0.68–4.31)		Ref	—	
High-risk group	4.41	(1.87–10.4)		1.91	(0.84–4.34)	
Intensive treatment	1.06	(0.76–1.46)	0.7	0.87	(0.66–1.15)	0.3

Anti-GBM, anti-glomerular basement membrane; AKRiS, improved ANCA kidney risk score; ARRS, ANCA renal risk score; C3 and C4, complement fractions 3 and 4; CRP, c-reactive protein; P/C ratio, urine protein-creatinine ratio.

Univariable analysis. Values are expressed as subdistribution hazard ratio (sHR) and 95% confidence interval (95%CI).

### Exploratory Analyses of Seronegative Anti-GBM Disease

Exploratory analyses showed that patients with seronegative anti-GBM disease exhibited less severe renal involvement upon presentation, characterized by a lower rate of oligoanuria (38% vs. 68% in classical anti-GBM; *P* = 0.01), a lower need for dialysis (52% vs. 77%; *P* = 0.02), and lower AKRiS scores (11 [6–18] vs. 18 [11–21]; *P* = 0.0003; Supplementary Table S6). The proportion of patients who remained alive and dialysis-free was also higher than that of patients with classical anti-GBM disease (43% vs. 20%, respectively; *P* = 0.02). In the multivariable Fine-Gray regression analysis, anti-GBM positivity was not associated with 36-month kidney failure.

## Discussion

This study shows the association of the improved AKRiS and kidney prognosis in a large cohort of patients with anti-GBM disease. Our findings suggest that the score stratifies the risk of 36-month kidney failure, maintaining its robustness regardless of ANCA status or standard-of-care treatment, such as PLEX.

As renal prognosis in anti-GBM disease is primarily established during the acute phase, early risk stratification is critical. In this study, AKRiS independently predicted 36-month kidney failure and demonstrated superior predictive performance compared with the older ARRS, reflecting how the refinement of continuous and categorical parameters enhances risk evaluation.^7^, ^19^, ^20^ However, the score's discriminative granularity was somewhat attenuated in our population—kidney failure incidence did not significantly differ between the low- and moderate-risk groups, or between the high- and very high-risk groups. This dichotomous separation likely reflects the marked severity and relative homogeneity of renal involvement at presentation in anti-GBM disease, where most patients already exhibit advanced kidney failure. To overcome this inherent bluntness, we demonstrated that combining AKRiS with a simple baseline functional parameter—oligoanuria—significantly improved its predictive accuracy. This enhancement was especially pronounced within the moderate-risk category, where the cumulative incidence of kidney failure ranged widely from 25% without oligoanuria to 75% with it.

Although other parameters (such as age, C-reactive protein and anti-GBM status) showed univariable associations, their inclusion did not yield any incremental improvement in the C-index and could introduce treatment-related biases. Thus, combining AKRiS—which accounts for both renal function and structural injury—with a clinical functional marker like oligoanuria yields a robust and parsimonious model for early risk stratification. Importantly, the predictive value of the composite model remained consistent even in the subgroup of patients receiving PLEX (*P* = 0.06) confirming that it captures baseline structural damage rather than just treatment exposure. Moreover, its performance in SPPs highlights the successful transposition of this score to a pure anti-GBM phenotype.

In clinical practice, this composite evaluation may help in distinguishing patients who demonstrate potential for kidney recovery from those exhibiting extensive, irreversible damage. For instance, of those who were oligoanuric and received intensive treatment in the high- and very high-risk groups, only 3 out of 38 (8%) and none out of 25 (0%) remained alive and kidney failure-free, respectively. Furthermore, of those requiring dialysis at presentation in these same groups, only 3 out of 52 (6%) and none of 40 (0%) of the oligoanuric patients recovered kidney function. These findings may support individualized discussions regarding the expected benefit-risk balance, as well as the intensity and duration of intensive immunosuppressive therapy, particularly in cases of kidney-limited disease. Nevertheless, it is important to note that AKRiS should not be used alone to justify treatment withdrawal, as renal recovery may still occur in a small subset of severely affected patients.^22^ This individualized approach is reflected in our cohort's treatment data; the lower cumulative doses of cyclophosphamide in the very high-risk group are likely a consequence of both the necessary pharmacological dose adaptations for severe renal impairment and physician treatment decisions in patients considered to have a limited likelihood of renal recovery. Conversely, a lower risk score suggests a greater potential for renal recovery, supporting the maintenance of maximal therapeutic effort.

Future studies should concentrate on the development and external validation of anti-GBM-specific prognostic models in larger, well-characterized, retrospective multicenter cohorts or collaborative registries. These models should integrate histopathologic findings with readily available clinical and biological parameters, such as oligoanuria. The use of composite models has the potential to more accurately capture the pathophysiology of anti-GBM disease, thereby facilitating more precise individualized risk assessments. The development of a robustly validated prognostic tool could enhance patient stratification in clinical trials, and optimize treatment strategies by identifying patients most likely to benefit from intensive immunosuppressive therapy while reducing unnecessary exposure to potentially toxic treatments in those with an extremely poor likelihood of renal recovery.

Consistent with the findings of previous studies, we found that DPPs were older and presented with more severe clinical and biological renal involvement, as reflected by higher SCr level, a higher prevalence of oligoanuria, and a greater need for dialysis. They also showed more chronic histologic lesions, including a higher proportion of glomerulosclerosis and more extensive IFTA. These lesions were associated with a reduced percentage of normal glomeruli, consequently leading to higher AKRiS scores.^4^, ^8^, ^23^ Despite this difference in AKRiS score, the cumulative incidence of 36-month kidney failure did not differ significantly from that of SPPs, aligning with a previous study by Hu *et al.*^9^ that reported similar long-term kidney outcomes between these 2 populations. This paradox suggests that SPPs may have a lower potential for renal recovery relative to their baseline injury characterized by rapidly destructive necrotizing lesions. Conversely, the ANCA-mediated component in DPPs may be more inflammatory and potentially more responsive to immunosuppression. Although the pathophysiology of DPPs remains incompletely understood, several mechanisms have been proposed. One leading hypothesis suggests that ANCA antibodies may precede the development of anti-GBM antibodies.^24^ In this model, ANCA-mediated glomerular injury has the potential to expose the normally sequestered GBM epitope [α3(IV)NC1], resulting in the loss of immune tolerance and the subsequent production of anti-GBM antibodies. This mechanism provides a rationale for the histopathologic features observed in DPPs, which frequently combine chronic and inflammatory lesions characteristic of ANCA-associated vasculitis. Furthermore, circulating anti-GBM antibody levels may influence the severity of the disease. Indeed, McAdoo *et al.*^6^ reported that DPPs generally exhibit lower anti-GBM antibody titers compared with SPPs suggesting this as a potential contributing factor to a less severe disease course and a greater responsiveness to immunosuppressive therapy. However, this observation was not confirmed in our cohort, where anti-GBM antibody levels did not differ significantly between DPPs and SPPs. Ultimately, AKRiS demonstrated a comparable ability to stratify kidney prognosis regardless of ANCA status, underscoring that established irreversible histologic damage drives the prognosis more than serological phenotypes. Thus, the apparently more severe presentation of DPPs should not discourage intensive treatment, as their intrinsic renal prognosis is not worse than that of SPPs.

Exploratory analyses of seronegative anti-GBM disease confirmed a less severe renal presentation and more favorable outcomes, with lower AKRiS values, and a higher proportion of dialysis-free survival. These findings are consistent with previous reports, including the review by Bharati *et al.*,^25^ which noted the less aggressive nature and better renal prognosis of atypical cases. However, anti-GBM seropositivity was not an independent predictor of kidney failure in our multivariable model, suggesting that baseline renal injury, rather than the serological status itself, primarily drives prognosis. The absence of circulating antibodies in our historical cohort can be partly explained by the lower sensitivity of the immunologic techniques available at the time.^26^ Although modern assays have a better diagnostic yield, a small subset of patients continues to exhibit seronegative results because of atypical antigenic targets or non-IgG subclasses.^27^, ^28^, ^29^ In this context, these cases highlight the crucial role of the pathologist—an early kidney biopsy remains essential not only to secure the diagnosis and guide prompt treatment in seronegative patients, but also to accurately stratify their risk of kidney failure using structural tools such as the AKRiS.

The present study has several limitations. First, its retrospective design may introduce potential data collection biases. Secondly, although this cohort is one of the largest reported for this rare condition, the number of DPPs remained limited, which may have reduced statistical power for specific subgroup analyses. Thirdly, the evaluation of kidney biopsies was performed locally at each center rather than centrally reviewed. Although this introduces potential inter-observer variability, it also reflects the real-world applicability of the score in routine clinical practice. Finally, the precise timing of dialysis initiation during the initial hospital stay was not documented in all patient records, precluding a more detailed assessment of its prognostic value. Despite these limitations, this study has notable strengths, primarily the analysis of a large multicenter cohort with a long median follow-up, providing a robust assessment of kidney outcomes in a disease where data are historically scarce.

In conclusion, this study shows that the AKRiS is associated with kidney outcomes in both SPPs and DPPs. Although it provides useful prognostic information regarding baseline damage, AKRiS should not be used as a standalone tool for clinical decision-making. Future collaborative studies integrating clinical parameters, such as oligoanuria, are needed to develop more robust composite models for anti-GBM disease.

## Disclosure

All the authors declared no competing interests.

## Supplementary Material

Supplementary File (PDF) **Figure S1.** The cumulative incidence curves of 36-month kidney failure stratified by AKRiS and oligoanuria status in Single Positive patients. Table S1. Patient clinical and biological characteristics at diagnosis. **Table S2.** Therapeutic management. **Table S3.** Patient outcomes based on AKRiS groups. **Table S4.** Factors associated with 36-month kidney failure in univariable and multivariable Fine-Gray analysis in patients receiving plasma exchanges. **Table S5.** Baseline characteristics according to ANCA status. **Table S6.** Patients characteristics according to serum anti-GBM status.

## Footnotes

Supplementary File (PDF)
**Figure S1.** The cumulative incidence curves of 36-month kidney failure stratified by AKRiS and oligoanuria status in Single Positive patients. **Table S1.** Patient clinical and biological characteristics at diagnosis. **Table S2.** Therapeutic management. **Table S3.** Patient outcomes based on AKRiS groups. **Table S4.** Factors associated with 36-month kidney failure in univariable and multivariable Fine-Gray analysis in patients receiving plasma exchanges. **Table S5.** Baseline characteristics according to ANCA status. **Table S6.** Patients characteristics according to serum anti-GBM status.
