# Supplementary material for: Association of the ANCA Kidney Risk Score With Kidney Failure in Anti-Glomerular Basement Membrane Disease
Source: Kidney Int Rep. 2026 Oct 6;11(12):107130. doi: 10.1016/j.ekir.2026.107130 (PMC13753012; doi:10.1016/j.ekir.2026.107130)
Supplement: Supplementary file 1 [file mmc1.pdf]

## Supplementary Material

**Supplementary Table S1.** Patient clinical and biological characteristics at diagnosis

**Supplementary Table S2**. Therapeutic management

**Supplementary Table S3.** Patient outcomes based on AKRiS groups

**Supplementary Table S4.** Factors associated with 36-month kidney failure in univariable and multivariable Fine-Gray analysis in patients receiving plasma exchanges

**Supplementary Table S5.** Baseline characteristics according to ANCA status

**Supplementary Table S6.** Patient characteristics according to serum anti-GBM status

**Supplementary Figure 1.** The cumulative incidence curves of 36-month kidney failure stratified by AKRiS and oligoanuria status in single-positive patients.

**Supplementary Table S1. Patient clinical and biological characteristics at diagnosis**

| | **Total** (n=163) | **Low risk** (n=20) | **Moderate risk** (n=31) | **High risk** (n=64) | **Very high risk** (n=48) | ***p*-value** |
|---|---|---|---|---|---|---|
| Age | 55 [30–69] | 38 [23–54] | 48 [23–69] | 57 [33–72] [b] | 65 [38–76] [c] | 0.004 |
| Women | 65 (40) | 7 (35) | 7 (23) | 27 (42) | 24 (50) | 0.10 |
| **Comorbidities** | | | | | | |
| Diabetes mellitus | 10 (6) | 0 (0) | 0 (0) | 4 (6) | 6 (13) | 0.08 |
| Chronic arterial hypertension | 58 (36) | 2 (10) | 11 (35) | 24 (38) | 21 (44) | 0.06 |
| Dyslipidemia | 33 (20) | 3 (15) | 7 (23) | 11 (17) | 12 (25) | 0.69 |
| Tobacco use | 65 (40) | 11 (55) | 16 (52) | 23 (36) | 15 (31) | 0.13 |
| Cardiovascular diseases | 12 (7) | 1 (5) | 4 (13) | 3 (5) | 4 (8) | 0.51 |
| Cancer | 20 (12) | 0 (0) | 1 (3) | 11 (17) | 8 (17) | 0.06 |
| Charlson Comorbidity Index | 1 [0–4] | 0 [0–1] | 2 [0–3] [a] | 1 [0–4] [b] | 3 [1–4] [c] | 0.001 |
| Performance status | 1 [1–2] | 1 [1–2] | 1 [1–2] | 1 [1–2] | 2 [1–2] [c,f] | 0.02 |
| **Circulating antibodies** | | | | | | |
| Anti–GBM | 142 (87) | 15 (75) | 23 (74) | 57 (89) | 47 (98) [c,e] | 0.006 |
| ANCA | 47 (29) | 0 (0) | 6 (19) [a] | 19 (30) [b] | 22 (46) [c,e] | 0.001 |
| MPO | 35 (74) | 0 (0) | 4 (67) | 15 (79) | 16 (73) | 0.80 |
| PR3 | 9 (19) | 0 (0) | 2 (33) | 2 (11) | 5 (31) | 0.39 |
| Antinuclear antibodies | 44 (27) | 6 (30) | 6 (19) | 20 (31) | 12 (25) | 0.64 |
| **Kidney involvement** | | | | | | |
| Oligoanuria | 104 (67) | 1 (5) | 11 (35) [a] | 52 (81) [b,d] | 40 (83) [c,e] | <0.0001 |

**Supplementary Table S1. Patient clinical and biological characteristics at diagnosis (continued)**

| | | | | | | |
|---|---|---|---|---|---|---|
| Arterial hypertension at presentation | 103 (66) | 9 (45) | 16 (52) | 42 (66) | 36 (75) | 0.05 |
| SCr (µmol/L) | 670 [388–1050] | 172 [80–253] | 365 [209–493] [a] | 960 [562–1270] [b,d] | 785 [622–1285] [c,e] | <0.0001 |
| SCr <250 µmol/L | 24 (15) | 15 (75) | 9 (29) [a] | 0 (0) [b,d] | 0 (0) [c,e] | <0.0001 |
| 250≤ SCr ≤450 µmol/L | 22 (13) | 5 (25) | 13 (42) | 4 (6) [b,d] | 0 (0) [c,e] | <0.0001 |
| SCr >450 µmol/L | 117 (72) | 0 (0) | 9 (28) [a] | 60 (94) [b,d] | 48 (100) [c,e] | <0.0001 |
| P/C ratio >3.0 g/g | 81 (50) | 9 (45) | 14 (45) | 36 (56) | 22 (46) | 0.61 |
| Required dialysis at diagnosis | 120 (74) | 3 (15) | 12 (39) | 58 (91) [b,d] | 47 (98) [c,e] | <0.0001 |
| **Pulmonary involvement** | | | | | | |
| Diffuse alveolar hemorrhage | 56 (34) | 7 (35) | 12 (39) | 21 (33) | 16 (33) | 0.95 |
| **Laboratory parameters** | | | | | | |
| Serum albumin (g/L) | 26.0 [22.5–29.8] | 25.8 [22.5–28.6] | 28.5 [23.0–34.0] | 25.0 [22.4–28.8] | 25.0 [22.0–28.4] | 0.21 |
| Leukocyte count (cells/mm$^3$) | 10.5 [7.9–12.9] | 11.2 [9.0–12.4] | 11.2 [7.6–12.3] | 10.2 [7.5–13.7] | 10.3 [8.0–13.2] | 0.92 |
| Hemoglobin (g/dL) | 8.6 [7.5–9.8] | 9.3 [7.5–11.7] | 8.5 [7.2–10.6] | 8.7 [7.5–9.9] | 8.3 [7.6–9.1] | 0.57 |
| C–reactive protein (mg/L) | 102 [34–182] | 38 [2–153] | 53 [14–151] | 120 [63–219] [b,d] | 104 [49–179] | 0.003 |
| C3 (g/L) | 1.22 [1.05–1.40] | 1.26 [1.08–1.51] | 1.23 [1.09–1.41] | 1.17 [1.02–1.35] | 1.23 [1.10–1.41] | 0.30 |
| C4 (g/L) | 0.29 [0.23–0.35] | 0.29 [0.19–0.40] | 0.27 [0.21–0.33] | 0.29 [0.23–0.36] | 0.30 [0.26–0.34] | 0.66 |

Values are expressed as the median [interquartile range] or the number (percentage). Anti-GBM, antibodies against glomerular basement membrane; ANCA, anti-neutrophil cytoplasmic antigen, MPO, myeloperoxidase; PR3, proteinase 3; SCr: serum creatinine; P/C ratio, urine protein-creatinine ratio; C3 and C4, complement fractions 3 and 4. [a] p<0.05 low- vs moderate-risk group, [b] p<0.05 low- vs high-risk group, [c] p<0.05 low- vs very high-risk group, [d] p<0.05 moderate- vs high-risk group, [e] p<0.05 moderate- vs very high-risk group, [f] p<0.05 high- vs very high-risk group.

**Supplementary Table S2. Therapeutic management**

| | **Total** (n=163) | **Low risk** (n=20) | **Moderate risk** (n=31) | **High risk** (n=64) | **Very high risk** (n=48) | ***p*-value** |
|---|---|---|---|---|---|---|
| Intensive treatment | 114 (70) | 15 (75) | 22 (71) | 45 (70) | 32 (67) | 0.92 |
| Methylprednisolone pulses | 162 (99) | 20 (100) | 30 (97) | 64 (100) | 48 (100) | 0.23 |
| | 3 [3–3] | 3 [3–3] | 3 [3–3] | 3 [3–3] | 3 [3–3] | 0.67 |
| CST at 3 months$ | 134 (87) | 19 (95) | 31 (100) | 50 (83) [d] | 34 (71) [e] | 0.03 |
| CST at 6 months$ | 97 (64) | 14 (70) | 27 (87) | 36 (60) [d] | 20 (50) [e] | 0.01 |
| CST daily 6-month dose (mg) | 10 [10–15] | 10 [8–20] | 10 [10–15] | 10 [10–15] | 10 [5–14] | 0.95 |
| CST at 12 months$ | 39 (26) | 8 (40) | 12 (39) | 13 (22) | 6 (15) | 0.05 |
| Plasma exchange | 127 (78) | 16 (80) | 24 (77) | 52 (81) | 35 (73) | 0.76 |
| Number of sessions | 10 [7–15] | 11 [9–14] | 12 [10–15] | 11 [7–19] | 10 [6–14] | 0.40 |
| Cyclophosphamide | 134 (82) | 17 (85) | 27 (87) | 53 (83) | 37 (77) | 0.42 |
| Cumulative 6-month dose (mg/kg) | 55 [30–91] | 81 [53–106] | 85 [35–119] | 47 [25–74] | 41 [25–62] [c,e] | 0.02 |
| Rituximab | 8 (5) | 1 (5) | 1 (3) | 4 (6) | 2 (4) | 0.92 |
| Co-trimoxazole prophylaxis | 110 (67) | 13 (65) | 23 (74) | 44 (69) | 30 (63) | 0.73 |

Values are expressed as the median [interquartile range] or the number (percentage). Anti-GBM, antibodies against glomerular basement membrane; CST, corticosteroids. $ reported as a percentage of alive patients (n=154 with 20 in the low-, 31 in the moderate-, 60 in the high- and 43 in the very high-risk groups at 3 months; n=151 with 20 in the low-, 31 in the moderate-, 60 in the high- and 40 in the very high-risk groups at 6 months, and n=150 with 20 in the low-, 31 in the moderate-, 59 in the high- and 40 in the very high- groups at 12 months). [a] p<0.05 low- vs moderate-risk group, [b] p<0.05 low- vs high-risk group, [c] p<0.05 low- vs very high-risk group, [d] p<0.05 moderate- vs high-risk group, [e] p<0.05 moderate- vs very high-risk group, [f] p<0.05 high- vs very high-risk group.

0

**Supplementary Table S3. Patient outcomes based on AKRiS groups**

| | Total (n=163) | Low risk (n=20) | Moderate risk (n=31) | High risk (n=64) | Very high risk (n=48) | *p*–value |
|---|---|---|---|---|---|---|
| **Death** | | | | | | |
| At 12 months | 12 (7) | 0 (0) | 0 (0) | 5 (8) | 7 (15) | 0.05 |
| At 36 months | 19 (12) | 1 (5) | 0 (0) | 7 (11) [d] | 11 (23) [e] | 0.01 |
| **Cumulative incidence of kidney failure (death as competing event)** | | | | | | |
| At 12 months | 76.7% [69.4-82.5] | 35.0% [15.1-55.8] | 38.7% [21.7-55.4] | 92.2% [81.6-96.8] | 97.9% [85.8-99.7] | |
| At 24 months | 77.9% [70.7-83.6] | 40.4% [18.8-61.2] | 41.9% [24.3-58.6] | 92.2% [81.6-96.8] | 97.9% [85.8-99.7] | |
| At 36 months | 77.9% [70.7-83.6] | 40.4% [18.8-61.2] | 41.9% [24.3-58.6] | 92.2% [81.6-96.8] | 97.9% [85.8-99.7] | |
| **eGFR in patients alive without kidney failure (dialyzed or transplanted)** | | | | | | |
| At 12 months n (%) | 44 (27) | 15 (75) | 19 (61) | 9 (14) [b,d] | 1 (2) [c,e,f] | <0.0001 |
| eGFR (ml/min) * | 36 [22–59] | 47 [22–83] | 45 [32–59] | 24 [12–51] | 20 | NA |
| At 36 months n (%) | 37 (23) | 12 (60) | 18 (58) | 6 (9) [b,d] | 1 (2) [c,e] | <0.0001 |
| eGFR (ml/min) § | 53 [31–87] | 63 [27–102] | 56 [42–87] | 32 [19–52] | 17 | NA |
| **Relapse during follow-up** | 12 (7) | 4 (20) | 4 (13) | 2 (3) | 2 (4) | NA |

eGFR, estimated glomerular filtration rate. NA, non-applicable. Cumulative incidence is presented with 95% confidence interval. With low-risk group as reference, high- and very-high risk presented significantly higher incidence of kidney failure at each time point (Wald test p value< 0.001). Statistical comparisons for eGFR during follow-up were not performed due to the limited sample size and the significant survivor bias in the high- and very high-risk groups. [a] p<0.05 low- vs moderate-risk group, [b] p<0.05 low- vs high-risk group, [c] p<0.05 low- vs very high-risk group, [d] p<0.05 moderate- vs high-risk group, [e] p<0.05 moderate- vs very high-risk group, [f] p<0.05 high vs very high-risk group. * n= 38 patients with available data (from 44 patients alive non-transplanted) (13 in the low-, 16 in the moderate-, 8 in the high-, and 1 in the very high-risk groups). § n= 34 patients with available data (from 37 patients alive non-transplanted) (10 in the low-, 18 in the moderate-, 5 in the high-, and 1 in the very high-risk groups).

**Supplementary Table S4. Factors associated with 36-month kidney failure in univariable and multivariable Fine-Gray analysis in patients receiving plasma exchanges**

| | Univariable analysis | | | Multivariable analysis | | |
|---|---|---|---|---|---|---|
| **Variables** | **sHR** | **[95% CI]** | **p-value** | **sHR** | **[95% CI]** | **p-value** |
| Age at diagnosis (year) | 1.01 | [1.00-1.01] | 0.007 | | | |
| Sex (Women) | 1.03 | [0.81-1.31] | 0.8 | | | |
| Oligoanuria | 2.06 | [1.47-2.87] | <0.001 | 1.49 | [1.05-2.10] | 0.03 |
| P/C ratio>3g/d | 1.12 | [0.88-1.42] | 0.3 | | | |
| C3 (g/L) | 0.84 | [0.57-1.23] | 0.4 | | | |
| C4 (g/L) | 1.23 | [0.30-5.10] | 0.8 | | | |
| CRP (mg/L) | 1.00 | [1.00-1.00] | 0.003 | | | |
| Positive serum ANCA | 1.09 | [0.85-1.42] | 0.5 | | | |
| Positive serum anti-GBM | 1.25 | [0.75-2.10] | 0.4 | | | |
| AKRIS | | | <0.001 | | | <0.001 |
| Low-risk group | — | — | | — | — | |
| Moderate-risk group | 1.21 | [0.55-2.65] | | 1.05 | [0.47- 2.35] | |
| High-risk group | 2.87 | [1.54-5.37] | | 2.21 | [1.08-4.49] | |
| Very-high risk group | 3.27 | [1.78-6.00] | | 2.52 | [1.26-5.06] | |
| ARRS | | | <0.001 | | | |
| Low-risk group | — | — | | | | |
| Medium-risk group | 1.59 | [0.61-4.16] | | | | |
| High-risk group | 3.49 | [1.44-8.48] | | | | |

Values are expressed as subdistribution hazard ratio (sHR) and 95% confidence interval [95%CI]. P/C ratio, urine protein-creatinine ratio; C3 and C4, complement fractions 3; CRP , c-reactive protein; ANCA, anti-neutrophil cytoplasmic antigen; Anti-GBM, antibodies against glomerular basement membrane; AKRiS, improved ANCA kidney risk score; ARRS, ANCA renal risk score.

**Supplementary Table S5. Baseline characteristics according to ANCA status**

| | **Total** (n=163) | **DPPs** (n=47) | **SPPs** (n=116) | ***p*-value** |
|---|---|---|---|---|
| Age | 55 [30–69] | 68 [61–76] | 45 [26–65] | <0.0001 |
| Women | 65 (40) | 22 (47) | 43 (37) | 0.25 |
| **Comorbidities** | | | | |
| Tobacco use | 65 (40) | 10 (21) | 55 (47) | 0.002 |
| Charlson Comorbidity Index | 1 [0–4] | 3 [2–4] | 1 [0–3] | <0.0001 |
| Performance status | 1 [1–2] | 2 [1–2] | 1 [1–2] | 0.03 |
| **Antibodies** | | | | |
| Anti–GBM | 142 (87) | 41 (87) | 101 (87) | 0.97 |
| ANCA | 47 (28) | 47 (100) | - | |
| MPO | 35 (74) | 35 (74) | - | |
| PR3 | 9 (19) | 9 (19) | - | |
| **Kidney involvement** | | | | |
| Oligoanuria | 104 (67) | 37 (79) | 67 (58) | 0.01 |
| Arterial hypertension at presentation | 103 (66) | 33 (70) | 70 (60) | 0.24 |
| SCr (µmol/L) | 670 [388–1050] | 880 [560–1240] | 578 [293–1000] | 0.002 |
| SCr <250 µmol/L | 24 (15) | 2 (4) | 22 (19) | 0.02 |
| 250≥ SCr ≥450 µmol/L | 22 (13) | 2 (4) | 20 (17) | 0.03 |
| SCr >450 µmol/L | 117 (72) | 43 (92) | 74 (64) | 0.0004 |
| Required dialysis at diagnosis | 120 (74) | 41 (87) | 79 (68) | 0.01 |
| **Diffuse alveolar hemorrhage** | 56 (34) | 13 (28) | 43 (37) | 0.25 |
| **Laboratory parameters** | | | | |
| Hemoglobin (g/dL) | 8.6 [7.5–9.8] | 8.8 [7.7–10.1] | 8.5 [7.4–9.7] | 0.29 |
| C-reactive protein (mg/L) | 102 [34–182] | 108 [60–185] | 96 [23–182] | 0.20 |
| C3 (g/L) | 1.22 [1.05–1.40] | 1.24 [1.04–1.40] | 1.21 [1.07–1.40] | 0.67 |
| C4 (g/L) | 0.29 [0.23–0.35] | 0.27 [0.23–0.34] | 0.30 [0.23–0.35] | 0.64 |
| **Kidney biopsy findings at diagnosis** | | | | |
| **Light microscopy** | 163 (100) | 47 (100) | 116 (100) | |
| Number of normal glomeruli % | 0 [0–14] | 0 [0–6] | 0 [0–18] | 0.03 |
| >25% normal glomeruli | 27 (17) | 3 (6) | 24 (21) | 0.03 |

| | | | | |
|---|---|---|---|---|
| 10–25% normal glomeruli | 22 (13) | 6 (13) | 16 (14) | 0.86 |
| <10% normal glomeruli | 114 (70) | 38 (81) | 76 (66) | 0.045 |
| Glomerulosclerosis (%) | 11 [0–40] | 33 [0–64] | 7 [0–29] | 0.002 |
| IFTA ≥25% | 64 (39) | 29 (62) | 35 (30) | 0.0002 |
| IFTA (%)* | 15 [0–40] | 30 [14–50] | 10 [0–25] | <0.0001 |
| **AKRiS** | 18 [11–21] | 18 [18–21] | 18 [9–18] | <0.0001 |
| **Risk group** | | | | |
| Low | 20 (12) | 0 (0) | 20 (17) | 0.002 |
| Moderate | 31 (19) | 6 (13) | 25 (22) | 0.20 |
| High | 64 (39) | 19 (40) | 45 (39) | 0.85 |
| Very high | 48 (30) | 22 (47) | 26 (22) | 0.002 |
| **Therapeutic management** | 163 (100) | 47 (100) | 116 (100) | |
| Intensive treatment | 114 (70) | 28 (60) | 86 (74) | 0.06 |
| Methylprednisolone pulses | 162 (99) | 47 (100) | 115 (99) | 0.51 |
| Plasma exchange | 127 (78) | 33 (70) | 94 (81) | 0.13 |
| Cyclophosphamide | 134 (82) | 37 (79) | 97 (84) | 0.46 |
| Rituximab | 9 (6) | 3 (6) | 6 (5) | 0.76 |
| **Outcomes** | | | | |
| Death at 36 months | 19 (12) | 4 (9) | 15 (13) | 0.43 |
| Dialysis at 36 months death censored | 73 (51) | 27 (63) | 46 (46) | 0.05 |
| Alive dialysis-free patients at 36 months | 37 (23) | 9 (19) | 28 (24) | 0.50 |
| eGFR (ml/min)§ | 53 [31–87] | 174 [126-225] | 124 [95-189] | 0.08 |
| **Relapse at 36 months** | 12 (7) | 3 (6) | 9 (8) | 0.76 |

Values are expressed as the median [interquartile range] or the number (percentage). DPPs, double-positive patients; SPPs, single-positive patients; ANCA, anti-neutrophil cytoplasmic antigen, MPO, myeloperoxidase; PR3, proteinase 3; SCr: serum creatinine; C3 and C4, complement fractions 3 and 4. IFTA, interstitial fibrosis or tubular atrophy; AKRiS, improved ANCA kidney risk score; eGFR, estimated glomerular filtration rate. * n=158 (112 patients in SPPs group and 46 patients in DPPs group) with the exact percentage of IFTA given on the kidney biopsy reports. The other 5 patients had an IFTA≥ mild to moderate, considered as ≥ 25%. § n= 34 patients with available data (from 37 patients alive non-transplanted) (8 patients in SPPs group and 26 patients in DPPs group).

**Supplementary Table S6. Patient characteristics according to serum anti-GBM status**

| | **Total** (n=163) | **Anti-GBM positive** (n=142) | **Anti-GBM negative** (n=21) | ***p*-value** |
|---|---|---|---|---|
| Age | 55 [30–69] | 58 [29–71] | 45 [32–59] | 0.22 |
| Women | 67 (41) | 61 (43) | 6 (29) | 0.21 |
| Tobacco use | 65 (40) | 55 (39) | 10 (48) | 0.44 |
| **Antibodies** | | | | |
| ANCA | 47 (28) | 41 (29) | 6 (29) | 0.98 |
| **Kidney involvement** | | | | |
| Oligoanuria | 104 (67) | 96 (68) | 8 (38) | 0.01 |
| Arterial hypertension at presentation | 103 (66) | 90 (63) | 13 (62) | 0.90 |
| SCr (µmol/L) | 670 [388–1050] | 700 [452–1046] | 365 [203–1296] | 0.06 |
| SCr >450 µmol/L | 117 (72) | 108 (76) | 9 (43) | 0.002 |
| Required dialysis at diagnosis | 120 (74) | 109 (77) | 11 (52) | 0.02 |
| **Diffuse alveolar hemorrhage** | 56 (34) | 52 (37) | 4 (19) | 0.11 |
| **Kidney biopsy findings at diagnosis** | | | | |
| **Light microscopy** | 163 (100) | 47 (100) | 116 (100) | |
| Number of normal glomeruli % | 0 [0–14] | 0 [0–12] | 10 [0–33] | 0.03 |
| <10% normal glomeruli | 114 (70) | 104 (73) | 10 (48) | 0.02 |
| IFTA ≥25% | 64 (39) | 59 (42) | 5 (24) | 0.15 |
| IFTA (%)* | 15 [0-40] | 15 [0–40] | 15 [0–20] | 0.27 |
| **AKRiS** | 18 [11–21] | 18 [11–21] | 11 [6–18] | 0.0003 |
| **Therapeutic management** | 163 (100) | 142 (100) | 116 (100) | |
| Intensive treatment | 114 (70) | 104 (73) | 10 (48) | 0.02 |
| Methylprednisolone pulses | 162 (99) | 141 (99) | 21 (100) | 0.70 |
| Plasma exchange | 127 (78) | 115 (81) | 12 (57) | 0.01 |
| Cyclophosphamide | 134 (82) | 116 (82) | 18 (86) | 0.65 |
| Rituximab | 9 (6) | 9 (6) | 0 (0) | 0.25 |
| **Outcomes** | | | | |
| Death at 36 months | 19 (12) | 17 (12) | 2 (10) | 0.74 |

| | | | | |
|---|---|---|---|---|
| Dialysis at 36 months death censored | 73 (51) | 67 (54) | 6 (32) | 0.07 |
| Alive dialysis-free patients at 36 months | 37 (23) | 28 (20) | 9 (43) | 0.02 |
| eGFR (ml/min)§ | 53 [31–87] | 51 [26-89] | 55 [40-74] | 0.78 |
| **Relapse during follow-up** | 12 (7) | 10 (7) | 2 (10) | 0.68 |

Values are expressed as the median [interquartile range] or the number (percentage). ANCA, anti-neutrophil cytoplasmic antigen; SCr: serum creatinine; IFTA, interstitial fibrosis or tubular atrophy; AKRiS, improved ANCA kidney risk score; eGFR, estimated glomerular filtration rate. * n=158 (138 patients in anti-GBM positive group and 20 patients in anti-GBM negative group) with the exact percentage of IFTA given on the kidney biopsy reports. § n= 34 patients with available data (from 37 patients alive non-transplanted) (25 patients in anti-GBM positive group and 9 patients in anti-GBM negative group).

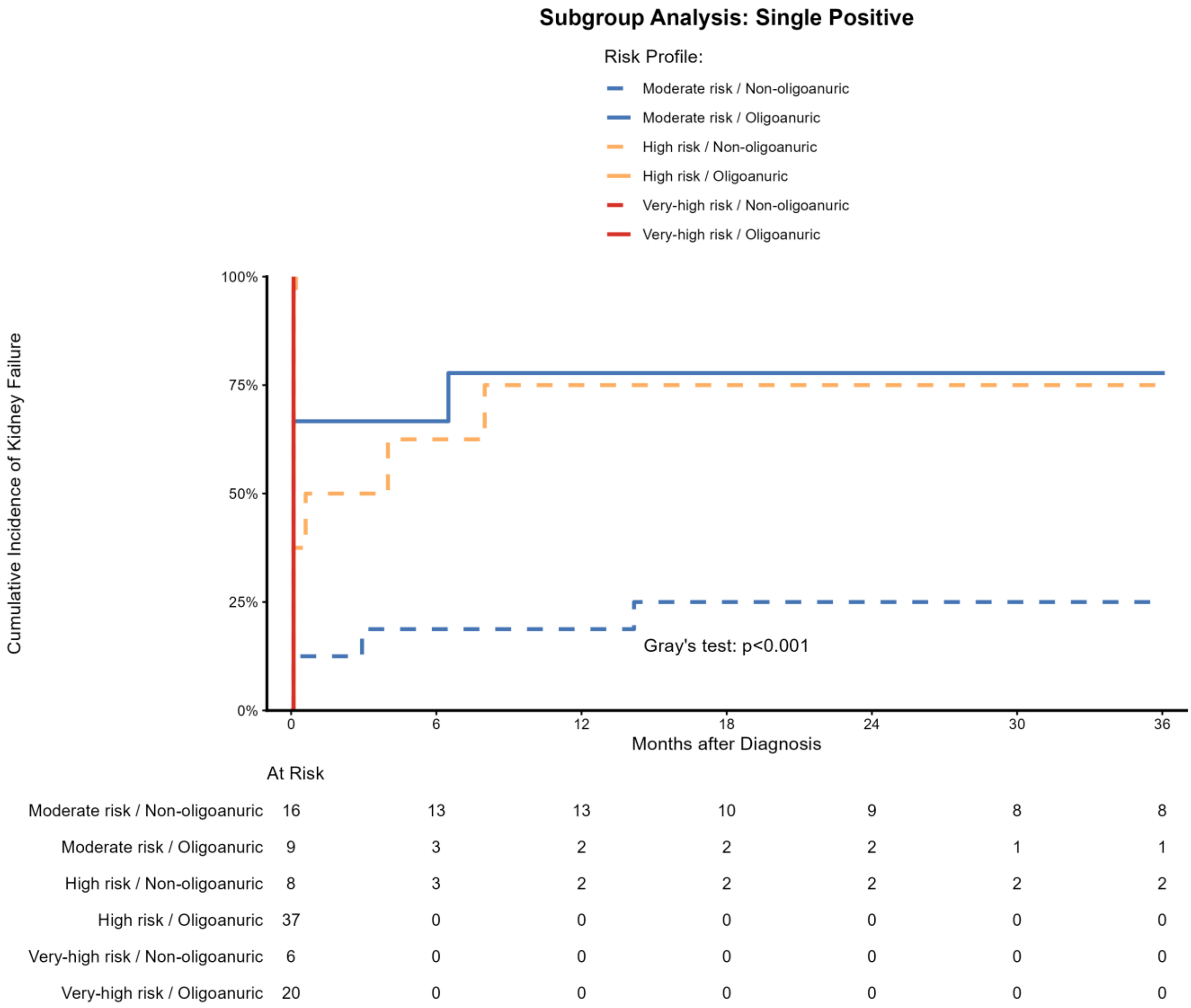


**Supplementary Figure 1. The cumulative incidence curves of 36-month kidney failure stratified by AKRiS and oligoanuria status in single-positive patients.** The curves illustrate the cumulative incidence of kidney failure in the single-positive patient group according to AKRiS class (moderate, high, and very-high) and oligoanuria status. Patients in the low-risk group were excluded from this analysis due to the presence of only one oligoanuric patient at baseline.
